# Natural menopause, menarche and breast cancer risk in *BRCA1* and *BRCA2* pathogenic variant carriers: a Mendelian randomization analysis

**DOI:** 10.1038/s41416-026-03365-6

**Published:** 2026-03-30

**Authors:** Nasim Mavaddat, Daniel R. Barnes, Kyriaki Michailidou, Emily Zhao, Debra Frost, Goska Leslie, Joe Dennis, Qin Wang, Manjeet K. Bolla, EMBRACE, Nasim Mavaddat, Emily Zhao, Debra Frost, Munaza Ahmed, Julian Barwell, Angela F. Brady, Paul Brennan, Hector Conti, Jackie Cook, Harriet Copeland, Rosemarie Davidson, Alan Donaldson, David Gallagher, Rachel Hart, Louise Izatt, Farah Kanani, Zoe Kemp, Fiona Lalloo, Zosia Miedzybrodzka, Patrick J. Morrison, Jennie E. Murray, Alex Murray, Hannah Musgrave, Claire Searle, Lucy Side, Katie Snape, Vishakha Tripathi, Lisa Walker, D. Gareth Evans, Marc Tischkowitz, Antonis C. Antoniou, Douglas F. Easton, D. Gareth Evans, Marc Tischkowitz, Deborah J. Thompson, John R. B. Perry, Antonis C. Antoniou, Douglas F. Easton

**Affiliations:** 1 Centre for Cancer Genetic Epidemiology, Department of Public Health and Primary Care, University of Cambridge, Cambridge, UK; 2 Department of Biostatistics, The Cyprus Institute of Neurology & Genetics, Nicosia, Cyprus; 3 Genomic Medicine, Manchester Academic Health Sciences Centre, Division of Evolution, infection and genomic science, University of Manchester, Manchester University Hospitals NHS Foundation Trust, Manchester, UK; 4 Department of Genomic Medicine, Cambridge Biomedical Research Centre, National Institute for Health Research, University of Cambridge, Cambridge, UK; 5 Metabolic Research Laboratory, Wellcome-MRC Institute of Metabolic Science, University of Cambridge School of Clinical Medicine, Cambridge, UK; 6 MRC Epidemiology Unit, Wellcome-MRC Institute of Metabolic Science, University of Cambridge School of Clinical Medicine, Cambridge, UK; 7 Centre for Cancer Genetic Epidemiology, Department of Oncology, University of Cambridge, Cambridge, UK; 8 North East Thames Regional Clinical Genetics Service, Great Ormond Street Hospital for Children NHS Trust, London, UK; 9 Leicestershire, Northamptonshire and Rutland Clinical Genetics Service, University Hospitals of Leicester NHS Trust, Leicester, UK; 10 North West Thames Regional Genetics Service, London North West University Healthcare NHS Trust, London, UK; 11 Northern Genetics Service, Newcastle Upon Tyne NHS Foundation Trust, Newcastle upon Tyne, UK; 12 All Wales Medical Genomics Services, Wrexham Maelor Hospital, Wrexham, UK; 13 Sheffield Clinical Genetics Service, Sheffield Children’s Hospital, Sheffield, UK; 14 Dept Clinical Genetics, Royal Devon University Healthcare NHS Foundation Trust, Exeter, Devon, UK; 15 Department of Clinical Genetics, South Glasgow University Hospitals, Glasgow, UK; 16 Clinical Genetics Department, St Michael’s Hospital, Bristol, UK; 17 Trinity St Jame’s Cancer Institute, Cancer Genetics Service, Dublin, Ireland; 18 Liverpool Women’s Hospital Cheshire and Merseyside Genetics, Liverpool Women’s NHS Foundation Trust, Liverpool, UK; 19 Clinical Genetics, Guy’s and St. Thomas’ NHS Foundation Trust, London, UK; 20 West Midlands Regional Clinical Genetics Service, Birmingham Women’s Hospital, Birmingham, UK; 21 Royal Marsden Hospital, NHS Trust, London, England UK; 22 Clinical Genetics Service, Manchester Centre for Genomic Medicine, Manchester University Hospitals Foundation Trust, Manchester, UK; 23 NHS Grampian, North of Scotland Regional Genetics Service, Aberdeen, Scotland UK; 24 Belfast Health and Social Care Trust, Clinical Genetics Service, Belfast, Northern Ireland UK; 25 South East Scotland Clinical Genetics Service, Western General Hospital, Edinburgh, UK; 26 All Wales Medical Genomics Service, Wales Genomic Health Centre, Cardiff, UK; 27 Leeds Genomic Medicine Service, Leeds, UK; 28 Department of Clinical Genetics, Nottingham University Hospitals NHS Trust, Nottingham, UK; 29 University Hospital Southampton NHS Trust and Princess Anne Hospital, Southampton, UK; 30 Medical Genetics Unit, St George’s, University of London, London, UK; 31 Oxford Centre for Genomic Medicine, Nuffield Orthopaedic Centre, Oxford University Hospitals NHS Foundation Trust, Oxford, UK

**Keywords:** Health sciences, Risk factors

## Abstract

**Background:**

The influence of age at menarche (AAM) and age at natural menopause (ANM) on breast cancer (BC) risk in *BRCA1* and *BRCA2* germline pathogenic variant (PV) carriers is uncertain. Observational studies are prone to bias and have limited statistical power. Mendelian randomization (MR) minimises bias and may be used to examine causal effects.

**Methods:**

Two-sample and age-specific MR analyses for BC were performed. For AAM, two-sample multivariable MR and mediation analyses to account for the confounding effect of body mass index (BMI), were undertaken.

**Results:**

Genetic scores for ANM and AAM predicted the respective traits in PV carriers. Inverse-variance weighted hazard ratios (HR) for genetically predicted ANM per-year were HR = 0.99 (95%CI:0.97–1.01, *p* = 0.45) and HR = 1.04 (95% CI:1.01–1.06, *p* = 0.003) for *BRCA1* and *BRCA2* PV carriers, respectively. After adjusting for genetic associations with BMI, AAM per-year on BC risk were HR = 0.90 (95%CI:0.83–0.98, *p* = 0.01) and HR = 0.95 (95%CI:0.86–1.04, *p* = 0.26) for *BRCA1* and *BRCA2* carriers, respectively, consistent with a protective effect of later AAM.

**Discussion:**

MR analyses support causal associations between ANM and BC risk in *BRCA2*, but not *BRCA1*, and between AAM and BC risk in *BRCA1* and *BRCA2* PV carriers. These results may aid risk prediction models and genetic counselling of carriers.

## Introduction

Early age at menarche (AAM) and late age at natural menopause (ANM) are established risk factors for breast cancer (BC) [1], attributed to extended exposure to steroid hormones that affect the development and function of the breast. A large overview of epidemiological studies reported a decrease in the relative risk of BC by approximately 5% for every year older at menarche [1]. Although the effect size per year is small, the range of risk can be considerable, and the decrease in the average age at menarche over time may have contributed to the increased disease incidence [2, 3]. The same overview also reported that delayed ANM increases the relative risk of overall BC by 3% for every year older at menopause, with the effect being stronger for ER-positive than ER-negative disease [1].

The extent to which these reproductive traits are associated with BC risk in *BRCA1* and *BRCA2* pathogenic variant (PV) carriers is less clear. Some investigators have reported an association between earlier AAM and increased BC risk in *BRCA1* PV carriers while reports for *BRCA2* PV carriers have been inconsistent [4–7]. Most epidemiological studies have been retrospective in design, and few large-scale prospective studies have been carried out in PV carriers. Furthermore, observational studies are prone to confounding. For example, there is a complex relationship between body mass index (BMI), AAM and BC risk, the influence of which may not have been adequately considered [8, 9]. Studies investigating an association between ANM and BC risk in PV carriers [10] are complicated by the fact that carriers frequently undergo prophylactic mastectomy and salpingo-oophorectomy (RRSO). Prospective studies therefore have low power, while the results of retrospective studies are difficult to interpret.

Mendelian randomization (MR) is a method to assess the association between an exposure and disease, using genetic markers associated with the exposure as instrumental variables (IV) [11, 12]. MR minimizes the effects of recall bias, reverse causation, measurement error, and residual confounding [13]. Key assumptions underlying MR are that genetic variants should be associated with the exposure of interest, should only affect the outcome through the exposure, and should not be associated with confounders in the exposure-outcome pathway [14]. While not all the MR assumptions are readily verifiable, the MR approach can strengthen the evidence for a causal relationship between exposure and disease.

Both AAM and ANM have strong genetic basis. Multiple independent AAM-associated single nucleotide polymorphisms (SNPs) have been discovered through genome-wide association studies (GWAS) [9, 15]. Many of these variants are also associated with BMI. ANM also has a strong genetic basis, with nearly 300 independent loci identified to date through GWAS [16]. SNPs associated with ANM are strongly enriched in DNA damage response pathways, including common coding variants in *BRCA1* [17]. Results from epidemiological studies comparing the distribution of ANM in PV carriers and non-carriers have, however, been conflicting [16, 18–21].

Here, we carried out MR analyses to investigate associations between SNP sets associated with AAM [9] and ANM [16], published by the Reprogen consortium. For AAM, we used two-sample multivariable MR to account for the mediating effect of BMI. We used data from a large national study to investigate whether genetic scores (GS) summarising the effect of common genetic variants associated with reproductive traits in the general population predict these traits in PV carriers, to confirm that these variants can be used as MR IVs for PV carriers. We then used GWAS summary statistics for associations between genetic variants and BC risk in PV carriers from the Consortium of Investigators of Modifiers of *BRCA1/2* (CIMBA) to carry out MR analyses, and compared the results with those from comparable analyses in the general population using data from the Breast Cancer Association Consortium (BCAC).

## Methods

### Study design and population (EMBRACE)

Participants were enrolled through an on-going prospective cohort study of individuals undergoing genetic testing at regional genomics centres in the United Kingdom and Ireland (Epidemiological Study of Familial Breast Cancer (EMBRACE)) (https://ccge.medschl.cam.ac.uk/embrace/). EMBRACE recruits individuals who are PV carriers in breast and/or ovarian cancer susceptibility genes, and their relatives. The analyses included only women of self-reported White ethnicity recruited between 1999 and 2023. Women were eligible if they were 18 years of age at recruitment and tested positive for a *BRCA1* or *BRCA2* PV, defined according to ENIGMA guidelines (https://clinicalgenome.org/affiliation/50087/).

### Data collection

Study participants completed a baseline questionnaire requesting detailed information on known or suspected breast and ovarian cancer risk factors, including family history of cancer, reproductive history and surgical interventions including risk-reducing mastectomy or RRSO. The questionnaires asked for information on age at last menstruation, whether the woman had had any period in the past year, the number of years/months since last menstruation, and reason(s) for periods stopping. Women were considered premenopausal if they indicated that they had had a period in the past year, or that their periods had not stopped completely, or if the ‘reason for periods stopping’ was medication or oral contraceptive use (unless 40 years or older), pregnancy, or breast-feeding, unless censored earlier due to cancer diagnosis, chemotherapy or radiotherapy, RRSO or hysterectomy. Age at menopause for those who indicated no period in the past year was determined by adding 1 year to ‘age at last menstruation’. Women were considered as having experienced natural menopause if reason for periods stopping was recorded as ‘natural menopause’ (and not for any other reason such as chemotherapy, childbirth, pregnancy, breast feeding, hysterectomy, or ‘unspecified’ reason) and if age at menopause preceded RRSO, any cancer diagnosis or interview. Women were also considered as menopausal at age 60 years. Women reporting RRSO or hysterectomy as the reason for periods stopping were considered premenopausal until age at last period. Women reporting periods stopping but with missing age at menopause or age at last period were excluded from the analyses. Age at menarche was coded as a continuous variable or categorised as age <12, 12–14 and ≥15 years (STable 1).

Genotypes on the EMBRACE participants were obtained using the iCOGS and OncoArray genome-wide assays, as part of reported analyses within the CIMBA consortium, followed by imputation using the 1000 Genomes Project reference panel (Phase 3) [22].

### Statistical analyses

To derive GS for ANM, we used uncorrelated SNPs associated with the trait at the genome-wide statistical significance level (*P* < 5 × 10^–8^), as identified by the Reprogen consortium [16]. Summary statistics for association with BC risk for 288 of the 290 SNPs were available. ANM SNPs were also categorized into those close to genes involved in DNA damage response (DDR) as previous defined (ANM signal ±300 kb within DDR and apoptosis genes) and all remaining SNPs (‘non-DDR’ SNPs) [16]. MR analyses were carried out separately on each SNP set. For 272 ANM SNPs, summary statistics for association with SHBG (adjusted for BMI) [23] were also available.

Summary statistics for association with BC for AAM SNPs were available for 350 of 377 autosomal SNPs at genome-wide statistical significance identified by Day et al. [9]. For 26 of the 350 loci, a surrogate variant (LD r^2^ > =0.8) was selected so that the corresponding effect sizes for association with BMI could be obtained. For 27 loci no suitable surrogate could be identified. The SNPs and corresponding weights for these analyses are shown in STables [additional STables ‘ANM input’ and ‘AAM input’].

While there is modest shared genome-wide genetic correlation between AAM and ANM [17] and regions of overlap have been identified [15], the large majority of IVs are uncorrelated between these traits, hence ANM-PGS and AAM-PGS were not jointly considered.

Sample sizes and studies are shown in STables 2–4. Genetic associations with ANM and AAM were based on ~ 200,000 [16] and 329,000 [9] women of European ancestry, respectively. Summary statistics from the meta-analysis of BMI in UK Biobank and GIANT consortium were based on ~800,000 individuals of European ancestry [24]. Summary statistics for SHBG were from UK Biobank based on 188,908 European ancestry women [23].

### Construction of genetic scores for reproductive factors

GS for ANM (ANM-GS) and AAM (AAM-GS) were constructed for a given individual as:

$${{{\rm{GS}}}}={{{{\rm{\beta }}}}}_{1}{\times }_{1}+{{{{\rm{\beta }}}}}_{2}\times _2+\ldots +{{{{\rm{\beta }}}}}_{{{{\rm{k}}}}}{{{{\rm{x}}}}}_{{{{\rm{k}}}}}\ldots .+{{{{\rm{\beta }}}}}_{{{n}}}{{{{\rm{x}}}}}_{{{n}}}$$

where β_k_ is the per-allele coefficient for association of the reproductive trait with SNP *k*, *x*_*k*_ is the allele dosage for SNP *k* and *n* is the total number of SNPs included in the score. The scores were standardized using their mean and standard deviation. A separate score based on AAM linked genetic variants weighted by BMI effect sizes (AAMBMI-GS) was also constructed.

### Testing validity of instrument variables in BRCA1 and BRCA2 PV carriers

Linear regression models allowing for a censored outcome were used to test the association between the standardized ANM score and age at menopause in EMBRACE, adjusting for birth cohort ( < 1940, 1940–1949, 1950–1959 and ≥1960). These analyses allowed data on both pre-menopausal and menopausal women to be included in the linear regression. Censoring was at the earliest of age at natural menopause, age at RRSO, any cancer diagnosis, death, age at interview or age 60 years. These models were undertaken using the VGAM R package. For comparison, ANM per unit SD of the GS in the general population was calculated using published SNP frequency and effect sizes, assuming independence between SNPs.

Linear regression models were also used to test for association between the standardised AAM-GS and AAM, adjusting for birth cohort ( < 1940, 1940–1949, 1950–1959, 1960–1969, ≥1970), and by BMI at age 18 years (kg/m^2^) or BMI at age at interview (kg/m^2^).

Robust variance estimates, clustering by family, were used to account for dependence among relatives. As participants were recruited from a population undergoing genetic testing, affected individuals are more likely to be sampled than unaffected individuals, and there is a higher probability of sampling younger affected individuals. To overcome this bias, a weighted cohort method was used, as described previously [25, 26]. Individuals were weighted such that the observed BC incidence rates were consistent with established age-specific incidence rates for *BRCA1* and *BRCA2* PV carriers [27–29] (STables 5 and 6).

### Breast cancer association statistics for Mendelian randomisation

Genetic associations between SNP genotypes and BC risk in *BRCA1* and *BRCA2* PV carriers, expressed as log(hazard ratio, HR) per allele, were obtained from GWAS analyses by CIMBA, based on 19,046 *BRCA1* and 12,412 *BRCA2* PV carriers [22] (https://www.ccge.medschl.cam.ac.uk/consortium-investigators-modifiers-brca12-cimba), (STable 2). HR estimates were obtained using a retrospective likelihood framework, as described previously [22, 25].

Age-specific HRs for each SNP and BC risk in CIMBA, were estimated using a weighted cohort approach [25, 26]. Weights for unaffected and affected PV carriers were calculated using birth cohort specific BC incidence rates [28] for eleven age categories (18–25, 25–30, …, 65–70, 70–80). Models were stratified by country, Ashkenazi Jewish ancestry, birth cohort ( < 1920, 1920–1929, …, 1970–1979, ≥1980) and the first four genotyping array-specific (iCOGS or OncoArray) principal components. Standard errors were obtained using a robust variance approach, clustering by family. Differences in the HR for BC risk by age were evaluated by fitting the main effects and interaction terms between the SNP dosages and age group ( < 40, 40,55) and ≥55. Sample sizes for these analyses are shown in STable 3.

For comparison, similar analyses were carried out using summary statistics for ER-negative and ER-positive BC risk in the general population, based on data from 122,977 cases (69,501 ER-positive and 21,468 ER-negative cases) and 105,974 controls of European ancestry from BCAC (https://www.ccge.medschl.cam.ac.uk/breast-cancer-association-consortium-bcac; STable 2) [22, 30]. Age specific odds ratios (ORs) ( < 40, 40-55 and >55 years) were derived as described in Michailidou et al.[30]. Sample sizes by age and ER-status for these analyses are shown in STable 4.

### Mendelian randomization

Instrumental variables for reproductive traits ANM and AAM were obtained as described above. Palindromic SNPs with allele frequency close to 0.5 (two ANM SNPs) were excluded.

For ANM we used the standard MR approach in which the effect size of each SNP for the association with BC is regressed on the effect size for association with the exposure (ANM). Thus:

$${\hat{\beta }}_{{YJ}}={\theta }_{T}{\hat{\beta }}_{{Xj}}+{\varepsilon }_{{Tj}},{\varepsilon }_{{Tj}} \sim N\left(0,{{{\rm{se}}}}{\left({\hat{\beta }}_{{Yj}}\right)}^{2}\right)$$

Where ${\hat{\beta }}_{{Yj}}$ is the per-allele log(HR) or log(OR) for BC risk associated with SNP *j*, with standard error ${se}({\hat{\beta }}_{{Yj}})$, ${\hat{\beta }}_{{Xj}}$, is the per-allele effect size for the exposure and ${\theta }_{T}$ is the effect of the exposure on outcome to be estimated. We focused on the inverse variance weighted estimate of the causal effect (IVW) but report the other standard estimates. In particular, we used MR Egger to assess for possible pleiotropic effects of the variants (where the effects are not mediated via the exposure), and carried out analyses removing outliers, using the R package RadialMR [31]. To address pleiotropy and the potential for genetic variants to influence BC risk both through the exposure traits and through pathways involving confounders related to these traits, we applied the pleiotropy-robust method MR-horse [32]. We also assessed the influence of sex hormone-binding globulin (SHBG), a transporter and regulator of androgens and oestrogen [33, 34], on the association between ANM and BC risk.

For AAM, two sample multivariable MR and mediation analysis to account for BMI was carried out as described in Burgess et al. [8]. Briefly, the total effect of the risk factor on the outcome was partitioned into a direct effect and a mediated effect due to BMI, by including an additional term in the regression to account for the BMI association:

$$\begin{array}{c}{\hat{\beta }}_{{YJ}}={\theta }_{{{{\rm{D}}}}}{\hat{\beta }}_{{XJ}}+{\theta }_{M}{\hat{\beta }}_{{MJ}}+{\varepsilon }_{{Dj}},{\varepsilon }_{{Dj}}\\ \sim NN\left(0,{{{\rm{se}}}}{\left({\hat{\beta }}_{{Yj}}\right)}^{2}\right)\end{array}$$

where ${\hat{\beta }}_{{MJ}}$ represents the per-allele effect for each SNP on BMI, ${\theta }_{D}$ is the direct effect of the exposure on risk (log(HR) per unit exposure) and ${\theta }_{M}$ is the mediated effect (log(HR) per unit BMI).

MR studies were reported according to standard guidelines [35, 36]. Statistical analyses were conducted using R version 4.3.1 and associated packages, VGAM [37, 38] https://CRAN.R-project.org/package=VGAM, tidyverse [39, 40], tidyr [41], Mendelian Randomisation (https://cran.r-project.org/web/packages/MendelianRandomization/index.html), meta [42], MR-horse [32], and radialMR [31]. Statistical tests were two-sided.

## Results

### EMBRACE study participants and characteristics

Data from EMBRACE was used to evaluate associations between genetic scores and AAM and ANM in *BRCA1* and *BRCA2* PV carriers. Genotype information was available for 1570 *BRCA1* and 1549 *BRCA2* PV carriers. A total of 183 (12%) *BRCA1* and 261 (17%) of *BRCA2* PV carriers experienced natural menopause prior to RRSO, a cancer diagnosis, or interview. Mean age at menarche was 13.0 and 12.9 years for *BRCA1* and *BRCA2* PV carriers, respectively. Cohort characteristics and the distribution of reproductive risk factors in EMBRACE are shown in STable 1.

### Genetic risk scores predict age at natural menopause and age at menarche in BRCA1 and BRCA2 PV carriers

Genetic scores based on SNPs and effect sizes estimated in the general population were highly predictive of ANM in *BRCA1* and *BRCA2* PV carriers, with higher ANM-GS associated with a later ANM (SFig. 1 and STables 7–9). In the censored linear regression analysis, each per SD increase in ANM-GS was associated with a 1.37 (95%CI:0.86–1.87, *p* = 1.1 × 10^–7^) years later ANM for *BRCA1* PV and 1.58 (95%CI:1.13–2.04, *p* = 6.7 × 10^–12^) years later ANM for *BRCA2* PV carriers (STable 9). The corresponding theoretical effect size in the general population, calculated using individual SNP effect sizes and allele frequencies, was 1.44.

The distributions of AAM-GS and AAMBMI-GS (AAM genetic variants weighted by BMI effect sizes) in PV carriers are shown in STable 10. In linear regression analyses, AAM-GS was predictive of AAM for *BRCA1* and *BRCA2* PV carriers (*p* = 1.2 × 10^–30^ and 4.2 × 10^–15^, respectively), with the mean of the standardised AAM-GS increasing with later age at menarche (STables 10 and 11). Age at menarche was 0.46 (95%CI: 0.39–0.54) and 0.33 (95%CI: 0.24–0.41) years later per unit SD of the AAM-GS in *BRCA1* and *BRCA2* carriers, respectively (STable 11). The corresponding theoretical effect size in the general population was 0.41.

### MR analysis of age at menopause on breast cancer risk

Of the 288 ANM variants associated with ANM in the general population, 29 and 16 respectively were nominally associated (*p* < 0.05) with BC risk for *BRCA1* and *BRCA2* PV carriers, respectively.

For *BRCA1*, the estimated IVW HR for genetically predicted ANM per year on BC risk was 0.99 (95%CI:0.97–1.01, *p* = 0.45). There was some evidence for heterogeneity by SNP (I^2^ = 26% [13.9%; 36.4%], Cochrane’s Q *p* = 7.1 × 10^–5^) (Table 1, SFig. 2). After removing outliers (29 SNPs), the IVW HR was unchanged with no evidence of association (HR = 1.00, 95%CI:0.98–1.02) and no evidence of heterogeneity (*p* > 0.05) (STable 12). In contrast, for *BRCA2* PV carriers genetically predicted ANM was positively associated with BC risk, (HR = 1.04, 95% CI:1.01–1.06, *p* = 0.003), with no evidence of heterogeneity (I^2^ = 0.0% [0.0%; 15.2%], *p* = 0.57) (Table 1, SFig. 2). Including SHBG in multivariable MR analyses made no difference to the association between ANM and BC in *BRCA1* and *BRCA2* PV carriers (Tables 1 and 2).

**Table 1:** Mendelian randomisation experiments assessing relationship between age at natural menopause and breast cancer risk in *BRCA1* and *BRCA2* PV carriers.

	*BRCA1* PV carriers				*BRCA2* PV carriers			
Method	HR	L95%	U95%CI	P-value	HR	L95%	U95%CI	*P* value
Simple median	0.99	0.96	1.02	0.51	1.04	1.00	1.08	0.04
Weighted median	1.00	0.97	1.04	0.92	1.03	0.99	1.08	0.11
Penalized weighted median	1.00	0.97	1.04	0.82	1.03	0.99	1.08	0.11
IVW	0.99	0.97	1.01	0.45	1.04	1.01	1.06	0.003
Penalized IVW	0.99	0.97	1.01	0.54	1.04	1.01	1.06	0.003
Robust IVW	0.99	0.97	1.02	0.59	1.04	1.02	1.06	0.001
Penalized robust IVW	1.00	0.98	1.02	0.62	1.04	1.02	1.06	0.001
MR-Egger	1.02	0.98	1.06	0.42	1.05	1.00	1.09	0.05
(intercept)	1.00	0.99	1.00	0.16	1.00	0.99	1.01	0.67
Penalized MR-Egger	1.02	0.98	1.06	0.30	1.05	1.00	1.10	0.04
(intercept)	1.00	0.99	1.00	0.13	1.00	0.99	1.01	0.55
Robust MR-Egger	1.02	0.98	1.06	0.29	1.05	1.00	1.09	0.05
(intercept)	1.00	0.99	1.00	0.12	1.00	0.99	1.01	0.71
Penalized robust MR-Egger	1.02	0.99	1.06	0.24	1.05	1.00	1.09	0.04
(intercept)	1.00	0.99	1.00	0.11	1.00	0.99	1.01	0.70
MR-horse	0.99	0.97	1.01		1.04	1.01	1.06	
MR-horse (adjusted for SHBG)	1.00	0.97	1.02		1.04	1.01	1.07	
Heterogeneity p-value:	7.1E-05				0.57			

Mendelian Randomization (MR) analyses to test the effect of later age at natural menopause (ANM) on breast cancer using a genetic instrument based on signals from the age at natural menopause genome-wide analysis as described in the Methods. Inverse-variance weighted (IVW) and other methods carried out as sensitivity analyses were used to generate effect estimates and P values (two-tailed). For some analyses the intercept is also shown in the rows below the estimates.

**Table 2:** Mendelian randomisation experiments assessing relationship between age at natural menopause and ER-positive and ER-negative breast cancer risk in the general population.

	ER-negative disease				ER-positive disease			
Method	OR	L95%	U95%CI	P value	OR	L95%	U95%CI	*P* value
Simple median	1.02	1.00	1.04	0.14	1.04	1.03	1.05	5.1E-08
Weighted median	1.03	1.01	1.05	0.002	1.05	1.03	1.06	4.9E-10
Penalized weighted median	1.03	1.01	1.05	0.003	1.05	1.04	1.07	6.4E-11
IVW	1.02	1.00	1.03	0.03	1.04	1.03	1.05	5.2E-09
Penalized IVW	1.02	1.01	1.03	0.004	1.05	1.04	1.06	1.1E-19
Robust IVW	1.02	1.01	1.04	0.01	1.05	1.03	1.06	5.5E-12
Penalized robust IVW	1.02	1.01	1.04	0.01	1.05	1.03	1.06	7.8E-14
MR-Egger	1.05	1.02	1.08	0.001	1.05	1.02	1.07	1.6E-04
(intercept)	1.00	0.99	1.00	0.018	1.00	1.00	1.00	0.36
Penalized MR-Egger	1.06	1.03	1.09	4.2E-06	1.05	1.03	1.07	3.0E-07
(intercept)	0.99	0.99	1.00	0.002	1.00	1.00	1.00	0.55
Robust MR-Egger	1.06	1.03	1.10	0.001	1.05	1.03	1.08	4.6E-06
(intercept)	0.99	0.99	1.00	0.02	1.00	1.00	1.00	0.37
Penalized robust MR-Egger	1.06	1.02	1.10	0.001	1.05	1.03	1.08	1.9E-05
(intercept)	0.99	0.99	1.00	0.02	1.00	1.00	1.00	0.56
MR-horse	1.02	1.01	1.04		1.05	1.03	1.06	
MR-horse (adjusted for SHBG)	1.02	1.01	1.04		1.05	1.04	1.06	
Heterogeneity p-value:	3.10E-13				3.06E-46			

Mendelian Randomization (MR) analyses to test the effect of later age at natural menopause (ANM) on breast cancer using a genetic instrument based on signals from the age at natural menopause genome-wide analysis as described in the Methods. Inverse-variance weighted (IVW) and other methods carried out as sensitivity analyses were used to generate effect estimates and P values (two-tailed). For some analyses the intercept is also shown in the rows below the estimates.

A range of sensitivity analyses were carried out to test validity and robustness to violations of the IV assumptions. Results from MR Egger, MR-horse and other analyses showed consistent effects (Table 1; STable 12).

MR analyses were also carried out using age-specific HRs for BC risk (STables 13 and 14). For *BRCA2* carriers there was some indication of an increasing HR by age, though the trend was not statistically significant (IVW estimate of 1.02 for women aged <40 years to 1.06 for women >=55 years). For *BRCA1* there was no evidence of association for any age-group.

Comparative analyses carried out for BC risk by ER-status and age in the general population (Table 2; STables 12–14) show a strong association with genetically predicted ANM for ER-positive disease (OR = 1.04, 95%CI:1.03–1.05, *p* = 5.2 × 10^–9^) and a weaker association for ER-negative disease (OR = 1.02, 95%CI:1.00–1.03, *p* = 0.03), p-diff = 0.03. The effect size showed an increasing trend in OR by age for both subtypes, with no association below age 40 years.

ANM SNPs were grouped into those located close to genes involved in DDR, and non-DDR SNPs (STables 15 and 16). In the general population, the estimated effect size for ER-positive disease was slightly larger for DDR-related SNPs, though the difference was not statistically significant (p-diff>0.05). For ER-negative disease a statistically significant association was only seen for DDR SNPs (p-diff=0.056). In *BRCA2* PV carriers, the effect size was similar for DDR and non-DDR SNPs. In *BRCA1* PV carriers, there was no association in either subset.

### MR analysis of age at menarche on breast cancer risk

Of the 350 AAM variants included in MR analyses, 20 and 24 were nominally associated (*p* < 0.05) with BC risk in *BRCA1* and *BRCA2* PV carriers respectively.

For *BRCA1* PV carriers, the estimated HR for the total effect of AAM on BC risk was 0.93 (95%CI:0.87–1.00, *p* = 0.03) per 1 year later menarche (Table 3, SFig. 3). After adjusting for genetic associations with BMI, the direct effect HR was 0.90 (95%CI:0.83–0.98, *p* = 0.01), consistent with a protective effect of later AAM. For *BRCA2* PV carriers the corresponding total and direct effects of AAM on BC risk were 0.99 (95%CI:0.91–1.07, *p* = 0.74) and 0.95 (95%CI:0.86–1.04, *p* = 0.26) (Table 3). There was no evidence of heterogeneity among SNPs. Results from MR Egger, MR horse and other analyses showed consistent effects and no evidence for horizontal pleiotropy (Table 3, SFig. 4).

**Table 3:** Mendelian randomisation experiments assessing relationship between age at menarche and breast cancer risk in *BRCA1* and *BRCA2* PV carriers.

	*BRCA1* PV carriers				*BRCA2* PV carriers			
Method	HR	L95%	U95%CI	P value	HR	L95%	U95%CI	*P* value
Total effect	0.93	0.87	1.00	0.03	0.99	0.91	1.07	0.74
Direct effects	0.90	0.83	0.98	0.01	0.95	0.86	1.04	0.27
Simple median	0.92	0.83	1.02	0.10	0.98	0.86	1.11	0.69
Weighted median	0.87	0.78	0.98	0.02	1.03	0.89	1.18	0.73
Penalized weighted median	0.89	0.79	0.99	0.04	1.04	0.90	1.20	0.57
IVW	0.90	0.83	0.98	0.01	0.95	0.86	1.04	0.26
Penalized IVW	0.92	0.85	0.99	0.02	0.96	0.87	1.06	0.40
Robust IVW	0.91	0.83	1.00	0.05	0.97	0.86	1.08	0.54
Penalized robust IVW	0.92	0.84	1.00	0.04	0.97	0.87	1.08	0.57
MR-Egger	0.88	0.76	1.02	0.08	1.00	0.84	1.20	0.99
(intercept)	1.00	1.00	1.01	0.67	1.00	0.99	1.00	0.46
Penalized MR-Egger	0.88	0.77	1.02	0.08	1.00	0.84	1.20	0.98
(intercept)	1.00	1.00	1.01	0.71	1.00	0.99	1.00	0.55
Robust MR-Egger	0.91	0.74	1.11	0.36	1.02	0.81	1.28	0.90
(intercept)	1.00	1.00	1.01	0.98	1.00	0.99	1.01	0.60
Penalized robust MR-Egger	0.91	0.75	1.11	0.34	1.02	0.81	1.27	0.89
(intercept)	1.00	1.00	1.01	0.99	1.00	0.99	1.01	0.61
MR-horse	0.90	0.83	0.98		0.94	0.85	1.04	

Mendelian Randomization (MR) analyses describing the total effect (first row) of age at menarche on breast cancer risk and the direct effect (second row) after adjusting for genetic associations with BMI from the multivariable analysis, as described in the Methods. Inverse-variance weighted (IVW) and other methods (adjusting for genetic associations with BMI) carried out as sensitivity analyses were used to generate effect estimates and P values (remaining rows, two-tailed). For some analyses the intercept is also shown in the rows below the estimates.

Similar analyses were carried out for ER-negative and ER-positive BC in the general population. For ER-negative disease ORs were 1.00 (95%CI:0.95–1.05) and 0.94 (95%CI:0.89–0.99, *p* = 0.02) and for ER-positive disease OR = 0.99 (95%CI:0.96–1.03) and 0.95 (95%CI:0.91-0.99, *p* = 0.02), for the total and direct effects of AAM respectively (Table 4, SFigs. 3 and 4).

**Table 4:** Mendelian randomisation experiments assessing relationship between age at menarche and ER-positive and ER-negative breast cancer risk in the general population.

	ER-negative disease				ER-positive disease			
Method	OR	L95%	U95%CI	P value	OR	L95%	U95%CI	*P* value
Total effect	1.00	0.95	1.05	0.99	0.99	0.96	1.03	0.76
Direct effect	0.94	0.89	0.99	0.02	0.95	0.91	0.99	0.02
Simple median	0.93	0.87	1.00	0.05	0.97	0.92	1.02	0.21
Weighted median	0.95	0.87	1.02	0.15	0.95	0.90	1.00	0.06
Penalized weighted median	0.95	0.88	1.02	0.16	0.96	0.91	1.01	0.10
IVW	0.94	0.89	0.99	0.02	0.95	0.91	0.99	0.02
Penalized IVW	0.95	0.91	1.00	0.05	0.96	0.93	1.00	0.05
Robust IVW	0.96	0.91	1.01	0.12	0.97	0.92	1.01	0.12
Penalized robust IVW	0.95	0.90	1.01	0.08	0.96	0.92	1.00	0.06
MR-Egger	0.92	0.83	1.02	0.12	0.88	0.81	0.96	0.004
(intercept)	1.00	1.00	1.00	0.70	1.00	1.00	1.01	0.05
Penalized MR-Egger	0.95	0.86	1.04	0.28	0.90	0.83	0.97	0.004
(intercept)	1.00	1.00	1.00	0.96	1.00	1.00	1.01	0.03
Robust MR-Egger	0.96	0.88	1.05	0.39	0.90	0.83	0.98	0.02
(intercept)	1.00	1.00	1.00	0.93	1.00	1.00	1.01	0.08
Penalized robust MR-Egger	0.96	0.88	1.04	0.31	0.90	0.83	0.97	0.006
(intercept)	1.00	1.00	1.00	0.93	1.00	1.00	1.01	0.05
MR-horse	0.95	0.90	1.00		0.96	0.92	1.00	

Mendelian Randomization (MR) analyses describing the total effect (first row) of age at menarche on breast cancer risk and the direct effect (second row) after adjusting for genetic associations with BMI from the multivariable analysis, as described in the Methods. Inverse-variance weighted (IVW) and other methods (adjusting for genetic associations with BMI) carried out as sensitivity analyses were used to generate effect estimates and P values (remaining rows, two-tailed). For some analyses the intercept is also shown in the rows below the estimates.

## Discussion

*BRCA1* and *BRCA2* PV carriers are at high risk of developing breast and ovarian cancer. However, this risk may be modified by other genetic, lifestyle and hormonal risk factors. We used Mendelian randomization to investigate causal relationships between ANM and AAM and BC risk in *BRCA1* and *BRCA2* PV carriers. Since genetic variants are inherited randomly at conception, MR provides a ‘natural experiment’ that avoids many of the pitfalls of observational studies. This is particularly valuable for evaluating risk factors in PV carriers, because prospective observational studies lack power as carriers are rare and many carriers undergo risk reducing surgery, while retrospective studies are subject to significant biases.

Using MR analyses, we showed that genetically predicted age at natural menopause influences BC risk in *BRCA2* carriers to a similar extent as found in the general population in both observational [1, 43, 44] and MR [17] analyses, with each one-year genetically predicted delay in ANM increasing the risk of developing *BRCA2* related BC by a factor of ~1.04. In contrast, we found no corresponding association in *BRCA1* carriers, and the 95% confidence limits (relative risk 0.97–1.01 per year) excluded the general population effect size (1.04, 95%CI: 1.03–1.05) [16]. This difference is plausible for two reasons. First, ANM may influence ER-positive disease risk more strongly than ER-negative and in particular, triple-negative disease [44, 45]. *BRCA1* PVs are predominately associated with triple-negative BC [46]. In addition, BC develops much earlier in *BRCA1* carriers—often below age 40 years—hence before natural menopause could influence risk. Breast cancer in *BRCA2* PV carriers occurs, on average, later and are associated to a greater extent with ER-positive disease.

In the age-specific analyses, however, ANM in *BRCA1* PV carriers was not associated with risk in any age-group, although the sample size at older ages was limited. These results are consistent with the observation that RRSO does not appear to reduce BC risk in *BRCA1* carriers [10]. BC incidence in *BRCA1* carriers is relatively constant beyond age 40, suggesting that most somatic events leading to tumorigenesis have already occurred by that point and disease risk is not subsequently influenced by hormonal changes.

Among *BRCA2* PV carriers, in contrast, the effect of delayed ANM was strongest for women aged 55 years or older (HR = 1.06). However, an association was observable even in the youngest age group ( < 40 years). This suggests that the association is not entirely due to menopause itself and that the influence of menopause-related biological processes on development of ER-positive BC could begin at a relatively early age. It is also possible that *BRCA2* carriers experience earlier ANM than women in the general population, though the evidence on this is so far unclear [47, 48].

For comparison, we examined the subtype-specific effects of genetically predicted ANM in the general population, using MR applied to data from the BCAC. The estimated ORs were 1.04 per year for ER-positive and 1.02 for ER-negative disease, consistent with an earlier publication on genetically predicted ANM (gANM) [17] and supporting subtype associations reported in some observational studies [1, 49], although a recent review of observational studies found inconclusive evidence for heterogeneity in ANM-associated risk by subtype [49]. In our age-specific MR analyses, there was some indication of a trend by age for ER-positive disease, with only a weak and non-significant effect below age 40 years, while the association for ER-negative disease was restricted to the 55+ years age-group. Taken together, the MR results in the general population are consistent with a stronger association for ER-positive BC and observed at older ages (i.e. after ANM, again consistent with a causal effect). For carriers, MR appear consistent with a model in which ANM influences BC to a similar relative extent in *BRCA2* carriers as in the general population, whereas there is little or no association in *BRCA1* carriers.

Menopause is intrinsically linked to the depletion of oocytes through DNA damage, and a substantial fraction of menopause associated SNPs are close to genes involved in DDR [16, 17, 50], a pathway in which *BRCA1* and *BRCA2* are crucial [51, 52]. Indeed, *BRCA1* is one of the GWAS associated loci for ANM. One might hypothesise that the IVs for ANM (and AAM) in the general population would not be valid in PV carriers, however we have shown that the ANM and AAM PGS also predict these traits in PV carriers to a similar extent. One might also expect some modulation of the ANM association according to the genetic mechanism. However, we saw no difference in the effect size for genetically predicted ANM on BC risk when SNPs were split into DDR and non-DDR categories. Better classification of SNPs may help refine these analyses and improve the power to evaluate interactions with BRCA status.

For AAM, in contrast, there was a clearer association with BC risk for *BRCA1* PV carriers. The ‘total’ causal effect (i.e. the predicted change in the outcome resulting from ‘intervening on the risk factor’), was 0.93 (95%CI:0.87–1.00) per 1 year later menarche. This total effect potentially encompasses multiple mediating mechanisms [8]. In particular, genetically predicted BMI is negatively associated with BC risk, and since BMI is a strong determinant of AAM, and genetically predicted BMI and genetically predicted AAM (gAAM) are correlated, this confounds the association between gAAM and BC. The ‘direct’ protective effect of AAM on BC risk in *BRCA1* carriers, after adjusting for BMI, was HR = 0.90 (95%CI:0.83–0.98, *p* = 0.01). The difference between the total and direct effect confirms that the causal effect of AAM on BC risk acts in part via BMI. The estimated HR were closer to 1 in *BRCA2* carriers (total effect HR = 0.99, 95%CI:0.91–1.07; direct effect HR = 0.95, 95%CI:0.86–1.04) but not significantly different from those in *BRCA1* effects.

In the general population, genetically predicted AAM was associated with both ER-negative and ER-positive disease, after controlling for genetically predicted BMI (OR = 0.94, *p* = 0.02 for ER-negative and OR = 0.95, *p* = 0.02 for ER-positive disease). These estimates are consistent with those from the PV carrier analyses, thus supporting a model in which AAM is associated with BC risk, both ER-negative and ER-positive disease, in the general population and in *BRCA1* and *BRCA2* PV carriers to a similar relative extent.

No statistically significant effect was found for ER-negative disease in the analysis by Day et al., but this is likely due to the smaller sample size of the outcome GWAS as the point estimates were similar [8, 9]. Observational studies have reported that later AAM was associated with reduced risk of all four intrinsic BC subtypes [45, 49], although the association was weakest for luminal B and HER2-overexpressing BC [53]. An association with ER-negative disease has also been reported in a study in African American women [54], and a recent review also supported no heterogeneity by ER-status [49].

While later AAM reduces sex-hormone exposure, the consistency of the associations by subtype and between PV carriers and general population suggest that other mechanisms may also influence risk. It has been proposed that rapid expansion of the breast epithelium during puberty creates a population of rapidly dividing cells susceptible to carcinogens [55, 56]. In vivo, oestrogen promotes both ER-negative and ER-positive *BRCA1*-deficient tumour initiation and progression [57].

Inherited *BRCA1* PVs lead to specific molecular and cellular alterations in breast epithelial differentiation before development of cancer, disease-free breast and ovarian tissues from *BRCA1* PV carriers exhibiting gene copy number gains and losses in key tumour suppressors and oncogenes [58]. It is plausible therefore that menarche and *BRCA1* carrier status could interact to increase BC risk. However, we did not find evidence for such an interaction.

For genetic variants to be a valid IV, they should be associated with the risk factor in question. Many of the SNPs associated with ANM are part of DNA damage repair (DDR) pathways (including common *BRCA1* and *BRCA2* variants), and their effect could be potentially enhanced (or diminished) on the background of impaired DNA repair. Further, studies in the UK Biobank suggest that rare protein truncating variants in *BRCA1* and *BRCA2* may be associated with earlier ANM, although these analyses would need to be replicated. Sample sizes in EMBRACE were insufficient to compare associations of individual SNPs in PV carriers. However, when combined as genetic scores, ANM-linked and AAM-linked variants predicted ANM and AAM respectively in *BRCA1* and *BRCA2* carriers from EMBRACE. Non-carriers were not genotyped in EMBRACE, so effect sizes in carriers and non-carriers could not be compared directly, however our effect estimates for genetic scores were broadly similar to those reported in the general population.

This study has some limitations. Known genetic variants explain a small proportion of the heritability of these traits. For ANM, 290 SNPs explain ~12% of the variance in ANM and 31–38% of the overall genotype-array estimated heritability [16]. For AAM, the 389 SNPs identified explain ~7.4% of the variance in AAM in the general population, corresponding to ~25% of the estimated heritability [9]. We carried out sensitivity analyses to test the validity of the causal inference and robustness to violations of the IV assumptions. However, some assumptions are not verifiable. For example, the restriction exclusion assumption cannot be directly tested. Associations between genetic variables and unmeasured confounders cannot be estimated. However, we applied a novel pleiotropy-robust method MR-horse to address potential for bias due to pleiotropy and violation of the instrument strength independent of direct effect (InSIDE) assumption. Causal inference using MR should be interpreted in the context of the specific assumptions that underlie MR analyses. Age-stratified analyses were limited in sample size, particularly for *BRCA1* PV carriers and at older ages. Subtype specific MR analyses in *BRCA1* and *BRCA2* carriers could be informative, but sample sizes were too limited for such analyses. Our analyses were carried out among women of European ancestry. While studies suggest a shared genetic basis of reproductive aging across populations of different ancestries [59], analyses evaluating causality non-European ancestries would be valuable. Finally, the potential role of hormonal factors such as SHBG and testosterone in BC causal pathways in carriers, in combination with ANM and AAM, should be explored in future analyses.

In conclusion, Mendelian randomization analyses indicate causal associations between ANM and BC risk in *BRCA2* PV carriers and AAM and BC risk in *BRCA1* and *BRCA2* PV carriers. Understanding risk factors and their effect sizes can aid risk prediction and management of PV carriers and can inform on mechanisms of BC carcinogenesis. From a practical viewpoint, these analyses suggest that assuming a similar relative risk in carriers to that in the general population, as is implemented in the BOADICEA risk prediction model [27], is a reasonable assumption for AAM, and for ANM in *BRCA2* PV carriers, but a specific adjustment may be needed for ANM in *BRCA1* carriers. There are clear advantages of MR in assessing the role of reproductive risk factors in BC aetiology. Nevertheless, large prospective cohort studies of PV carriers, with sufficient follow-up time and accurate information on surgeries and outcome, could provide independent confirmation of these findings.

## Supplementary information

Supplemental Material

Supplementary Tables

## Data Availability

The EMBRACE dataset generated and/or analysed during the current study are not publicly available, as they potentially include personal data. However, they can be accessed upon reasonable request made to the EMBRACE study Data Access Coordination Committee (embrace@medschl.cam.ac.uk) and the completion of a data sharing agreement. The BCAC data analysed in the current study are available upon request and approval from the BCAC Data Access Coordinating Committee (https://www.ccge.medschl.cam.ac.uk/breast-cancer-association-consortium-bcac/data-data-access). Access to individual level CIMBA data is governed by the CIMBA Data Access Coordinating Committee (https://www.ccge.medschl.cam.ac.uk/consortium-investigators-modifiers-brca12-cimba/data-data-access). Summary-level genotype data are available via https://www.ccge.medschl.cam.ac.uk/breast-cancer-association-consortium-bcac/data-data-access/summary-results and https://www.ccge.medschl.cam.ac.uk/consortium-investigators-modifiers-brca12-cimba/data-data-access/summary-results.
