# Supplementary material for: Natural menopause, menarche and breast cancer risk in BRCA1 and BRCA2 pathogenic variant carriers: a Mendelian randomization analysis
Source: Br J Cancer. 2026 Mar 30;134(10):1479–87. doi: 10.1038/s41416-026-03365-6 (PMC13133355; doi:10.1038/s41416-026-03365-6)
Supplement: Supplementary file 1 — Supplemental Material [file 41416_2026_3365_MOESM1_ESM.pdf]

Supplementary Information

Supplementary Tables 1-16 include Tables describing characteristics of *BRCA1* and *BRCA2* PV carriers, association between ANM and AAM genetic scores and the respective trait, studies and sample sizes included in MR, and results of MR of ANM and BC in different age-categories. Supplementary Tables 'ANM input' and 'AAM input' provide the SNPs and corresponding weights for both trait and outcome variables. Supplementary Figures SFigure 1-4 and Supplementary Figure Legends are shown below. A full list of EMBRACE centres and Principal investigators and the STROBE MR checklist are also included in this Supplementary file.

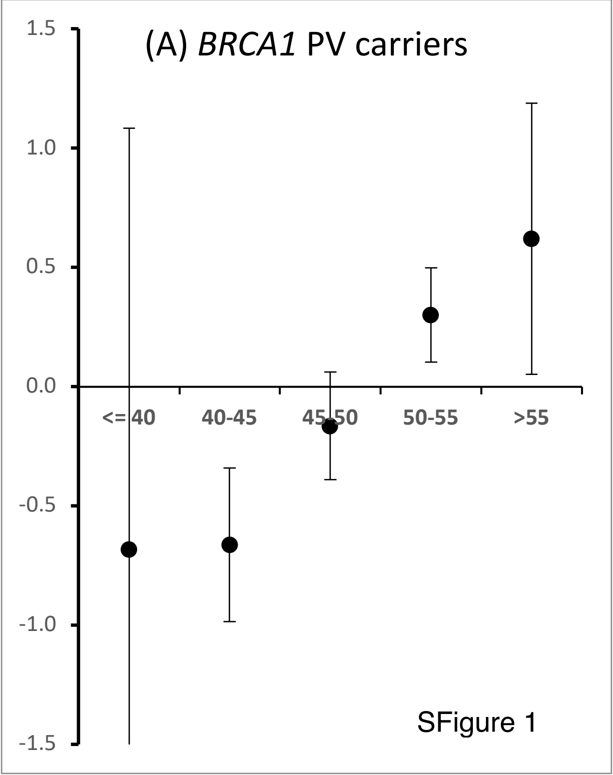

(A) BRCA1 PV carriers
1.5
1.0
0.5
0.0
-0.5
-1.0
-1.5
<= 40
40-45
45-50
50-55
>55
SFigure 1

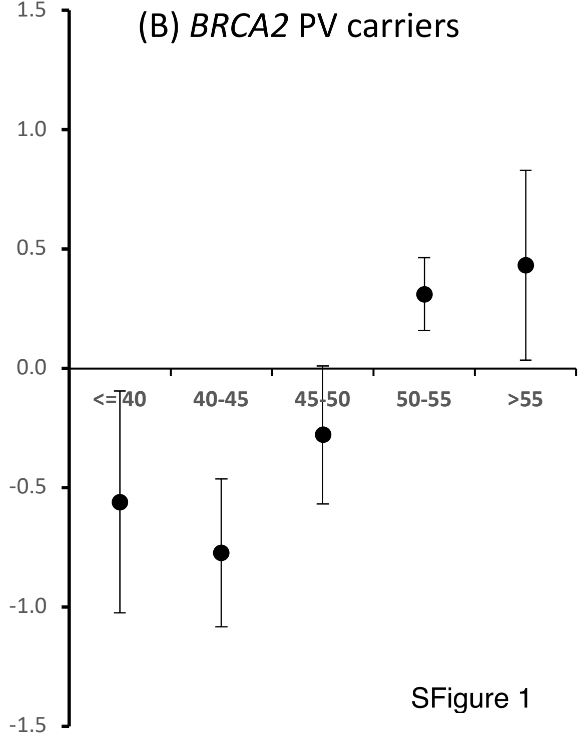

(B) BRCA2 PV carriers
1.5
1.0
0.5
0.0
-0.5
-1.0
-1.5
<=40
40-45
45-50
50-55
>55
SFigure 1

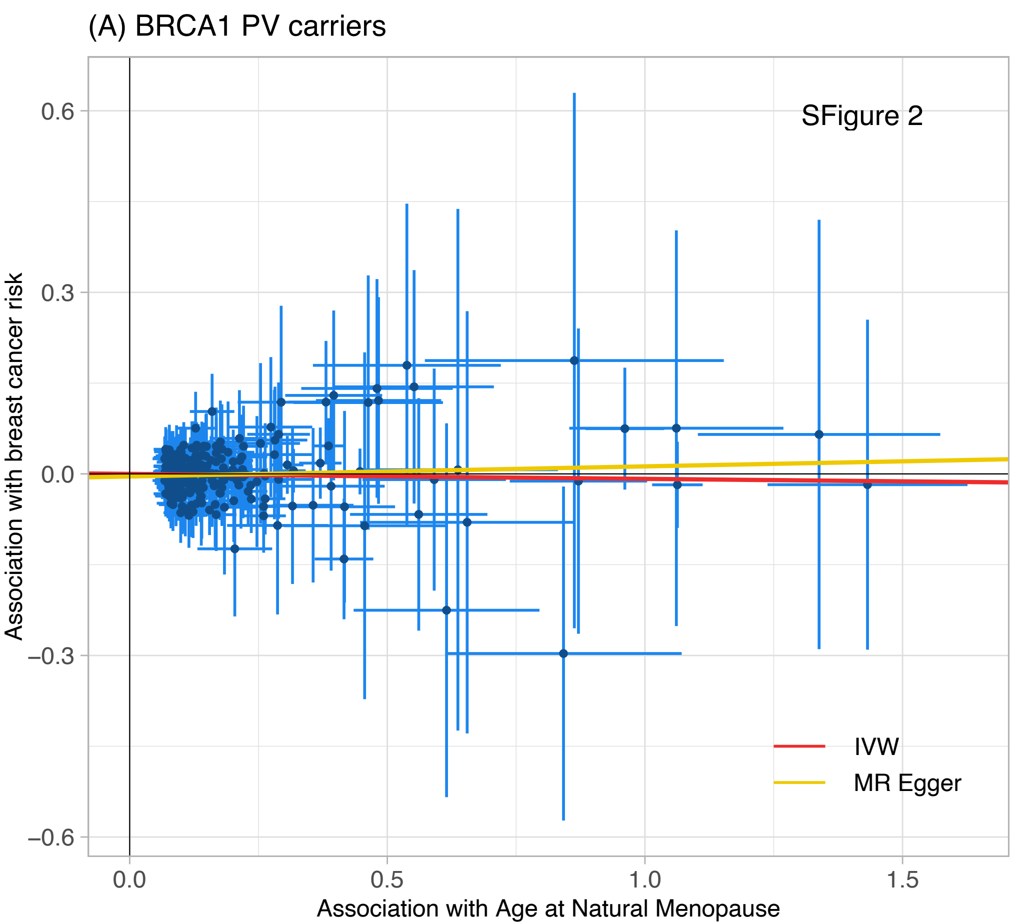
(A) BRCA1 PV carriers
SFigure 2
Association with breast cancer risk
0.6
0.3
0.0
−0.3
−0.6
0.0
0.5
1.0
1.5
Association with Age at Natural Menopause
IVW
MR Egger

(B) BRCA2 PV carriers

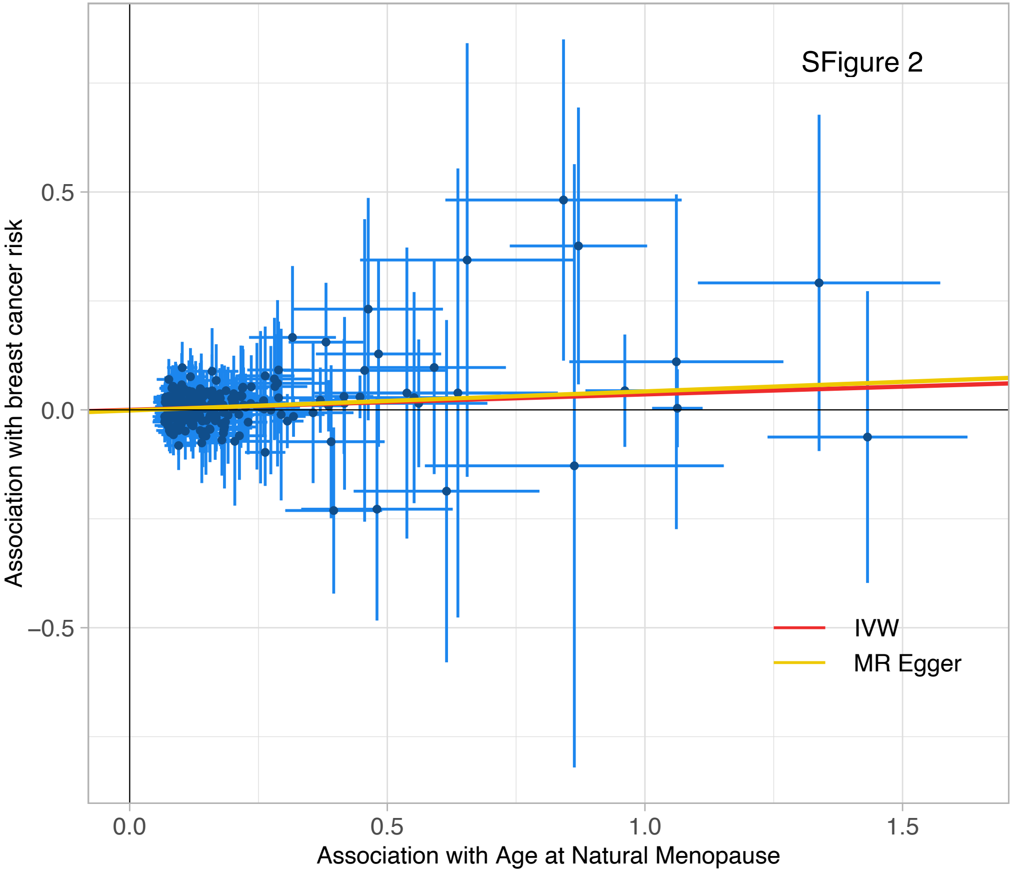

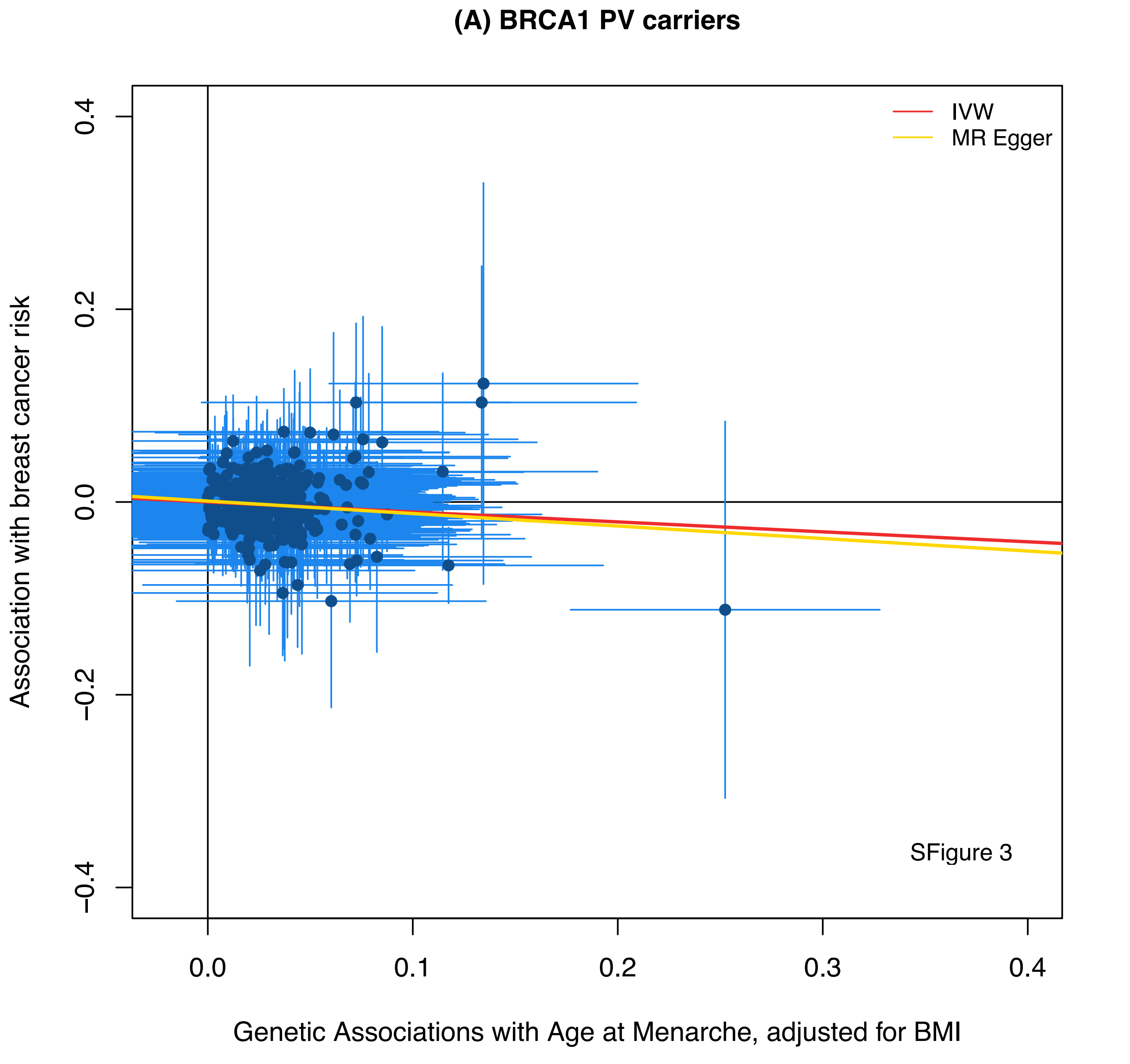
(A) BRCA1 PV carriers
IVW
MR Egger
SFigure 3
Association with breast cancer risk
Genetic Associations with Age at Menarche, adjusted for BMI
0.4
0.2
0.0
−0.2
−0.4
0.0
0.1
0.2
0.3
0.4

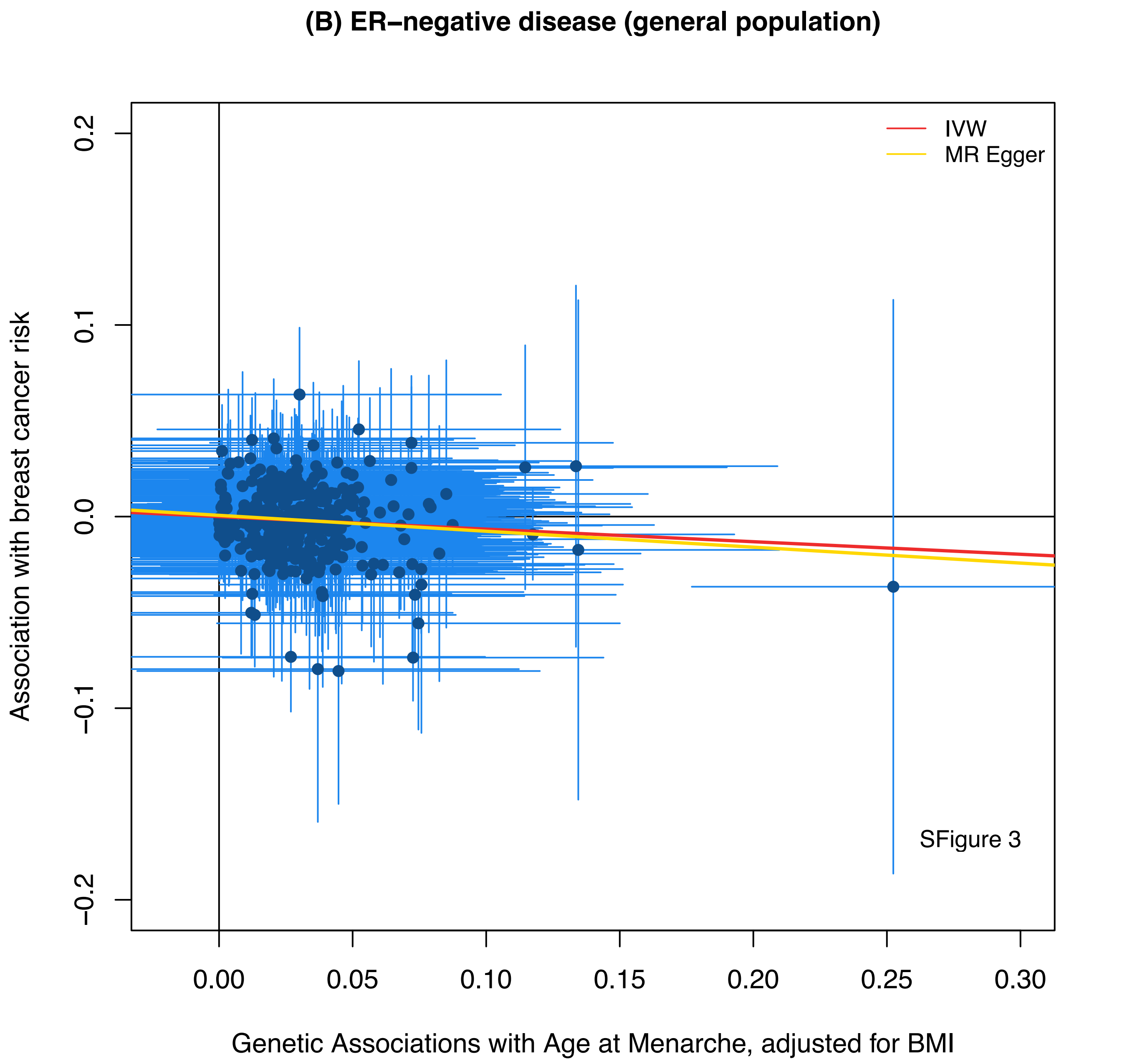

(B) ER−negative disease (general population)
IVW
MR Egger
SFigure 3
Association with breast cancer risk
−0.2
−0.1
0.0
0.1
0.2
0.00
0.05
0.10
0.15
0.20
0.25
0.30
Genetic Associations with Age at Menarche, adjusted for BMI

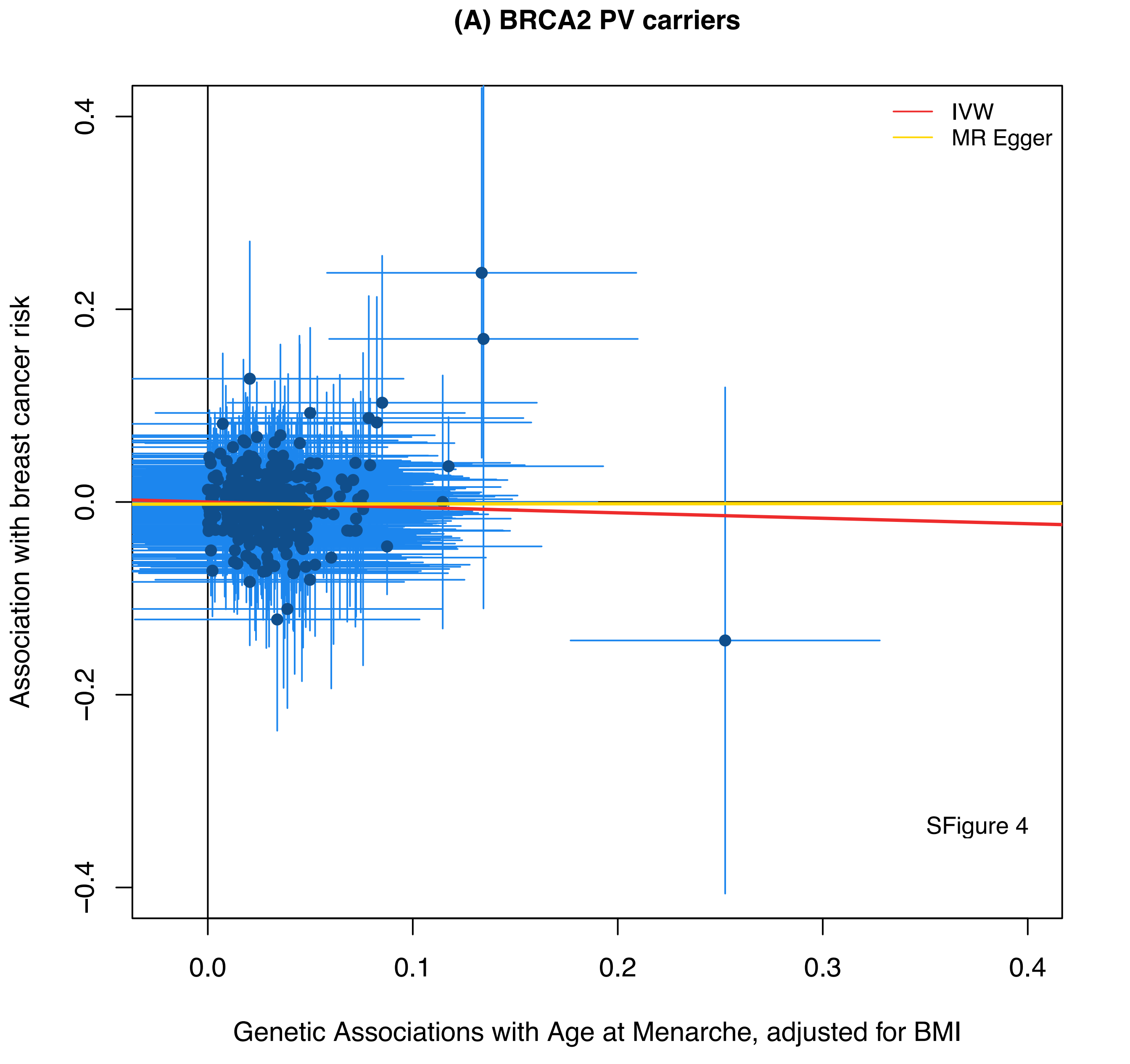

(A) BRCA2 PV carriers
IVW
MR Egger
SFigure 4
Association with breast cancer risk
0.4
0.2
0.0
−0.2
−0.4
0.0
0.1
0.2
0.3
0.4
Genetic Associations with Age at Menarche, adjusted for BMI

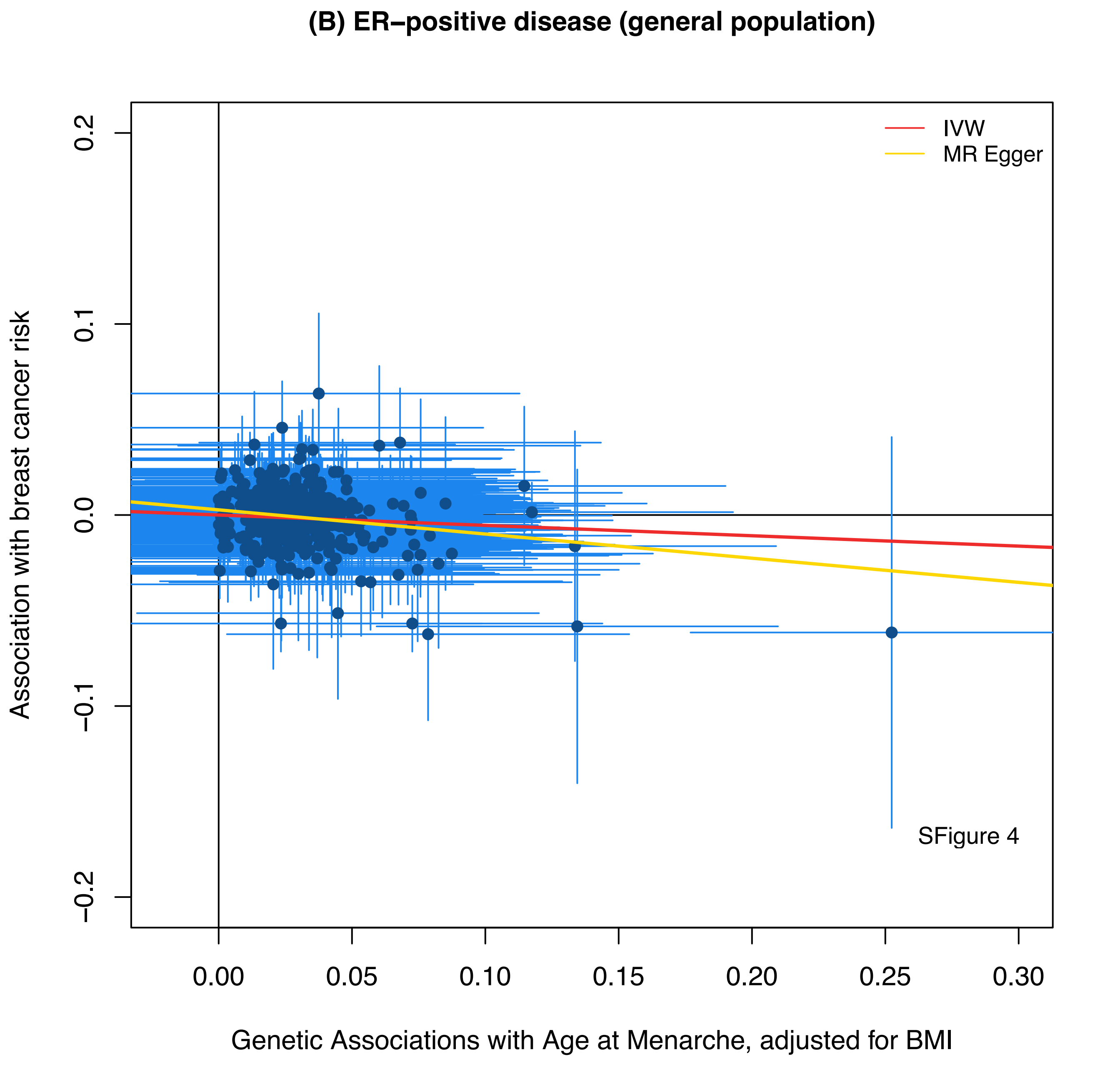

(B) ER−positive disease (general population)
IVW
MR Egger
SFigure 4
Association with breast cancer risk
Genetic Associations with Age at Menarche, adjusted for BMI
0.2
0.1
0.0
−0.1
−0.2
0.00
0.05
0.10
0.15
0.20
0.25
0.30

Supplementary Figure Legends:

SFigure 1. Polygenic prediction of age at natural menopause (ANM) for (A) *BRCA1* PV carriers and (B) *BRCA2* PV carriers. Mean sANM-GS is shown at different age categories of age at natural menopause. Higher mean ANM polygenic score indicates later ANM. The age at natural menopause genetic score (ANM-GS) was constructed using genetic variants discovered in the general population and scaled to have mean = 0 and standard deviation = 1, as described in Methods). These results are tabulated in STable 8.

SFigure 2. Genetic associations with age at menopause, plotted against genetic association with breast cancer among (A) *BRCA1* PV carriers and (B) BRCA2 PV carriers. IVW (red) and MR Egger (gold) estimates. Error bars for genetic associations are 95% confidence intervals.

SFigure 3. Genetic associations with age at menarche, after adjusting for BMI plotted against genetic association with breast cancer among (A) *BRCA1* PV carriers and (B) ER-negative breast cancers in the general population. IVW (red) and MR Egger (gold) estimates. Error bars for genetic associations are 95% confidence intervals.

SFigure 4. Genetic associations with age at menarche, after adjusting for BMI plotted against genetic association with breast cancer among (A) *BRCA2* PV carriers and (B) ER-positive breast cancers in the general population. IVW (red) and MR Egger (gold) estimates. Error bars for genetic associations are 95% confidence intervals.

Supplementary Table Legends:

STable 1: Characteristics of *BRCA1* and *BRCA2* PV carriers genotyped in CIMBA.

STable 2: Studies and sample sizes used in Mendelian Randomization analyses.

STable 3: Sample sizes for analyses of association between ANM SNPs and breast cancer risk by age at diagnosis/interview in CIMBA.

STable 4: Sample sizes for analyses of association between ANM SNPs and ER-negative or ER-positive disease by age at diagnosis/interview in BCAC.

STable 5: Computed weights in EMBRACE, assuming UK *BRCA1* and *BRCA2* PV carrier incidence rates.

STable 6: Breast cancer specific UK incidence rates used for estimation of weights.

STable 7: Distribution of the age at menopause genetic score among *BRCA1* and *BRCA2* PV carriers.

STable 8: Distribution of the standardised age at menopause genetic score (sANM-GS) by age at natural menopause.

STable 9: Association between the standardised age at menopause genetic score (sANM-GS) and age at natural menopause in *BRCA1* and *BRCA2* PV carriers.

STable 10: Distribution of the age at menarche genetic score (AAM-GS) and AAM variants weighted by BMI effect sizes (AAMBMI-GS) by categories of age at menarche.

STable 11: Association between the standardised age at menarche genetic score (sAAM-GS) and age at menarche in *BRCA1* and *BRCA2 PV* carriers.

STable 12: Mendelian randomisation experiments assessing relationship between ANM and breast cancer risk after removing variants identified as outliers using Radial MR.

STable 13: Mendelian randomisation experiments assessing relationship between age at natural menopause and breast cancer risk at different age categories.

STable 14: Mendelian randomisation experiments assessing relationship between ANM and breast cancer risk by age categories after removing variants identified as outliers using Radial MR.

STable 15: Mendelian randomisation experiments assessing relationship between ANM and breast cancer risk using all SNPs putatively involved in DDR mechanism, and all other SNPs.

STable 16: Mendelian randomisation experiments assessing relationship between ANM and breast cancer risk using all SNPs putatively involved in DDR mechanism and all other SNPs after removing variants identified as outliers using Radial MR.

Embrace Trusts and Principal Investigators

A full list of EMBRACE Trusts and Principal investigators follows: University Hospitals Bristol NHS Foundation Trust, Clinical Genetics, St Michael's Hospital: Alan Donaldson; All Wales Medical Genomics Service, Cardiff: Alex Murray; London North West Healthcare NHS Trust, North West Regional Genetics Service: Angela Brady; Nottingham University Hospitals NHS Trust, Clinical Genetics Service, City Hospital Campus: Claire Searle; Trinity St Jame's Cancer Institute, Cancer Genetics Service: David Gallagher; Birmingham Women's Health Care NHS Trust, West Midlands Regional Genetics Service: Farah Kanani; Manchester University NHS Foundation Trust, Manchester Centre for Genomic Medicine, St Mary's Hospital: Gareth Evans, Fiona Lalloo; Leeds Genomic Medicine Service, Yorkshire Regional Genetics Service: Hannah Musgrave; Royal Devon University Healthcare NHS Foundation Trust, Clinical Genetics Dept: Harriet Copeland; All Wales Medical Genomics Service, Cardiff: Hector Conti; Sheffield Children's NHS Foundation Trust, Sheffield Clinical Genetics Service: Jackie Cook; The Lothian University Hospitals NHS Trust, South East of Scotland Clinical Genetic Service: Jennie Murray; University Hospitals of Leicester NHS Trust, Dept of Clinical Genetics, Leicester Royal Infirmary: Julian Barwell; South West Thames Regional Genetics Service, St Georges University of London: Katie Snape; Oxford University Hospitals NHS Foundation Trust, Oxford Regional Genetics Service: Lisa Walker; Guy's and St Thomas' NHS Foundation Trust, Genetics Research Team, Guys Hospital: Louise Izatt, Vishakha Tripathi; University Hospital Southampton NHS Foundation Trust, Wessex Clinical Genetics Service: Lucy Side; Cambridge University Hospitals NHS Foundation Trust, East Anglian Medical Genetics Service, Addenbrookes Hospital: Marc Tischkowitz; Great Ormond Street NHS Hospital for Children NHS Foundation Trust, North East Thames Regional Genetics Service: Munaza Ahmed; Belfast Health and Social Care Trust, Clinical Genetics Service: Patrick Morrison; The Newcastle upon Tyne Hospitals NHS Foundation Trust, Northern Genetics Service: Paul Brennan; Liverpool Women's NHS Foundation Trust, Liverpool Centre for Genomic Medicine: Rachel Hart; NHS Greater Glasgow and Clyde, West of Scotland Genetics Services: Rosemarie Davidson; The Royal Marsden NHS Foundation Trust, Royal Marsden Clinical Genetics Unit: Zoe Kemp; NHS Grampian, North of Scotland Regional Genetics Service: Zosia Miedzybrodzka.

STROBE-MR checklist of recommended items to address in reports of Mendelian randomization studies[1 2]

Item No.	Section		Checklist item	Page No.	Relevant text from manuscript
1	**TITLE and ABSTRACT**		Indicate Mendelian randomization (MR) as the study's design in the title and/or the abstract if that is a main purpose of the study	✓ pp. 1, 3	
	**INTRODUCTION**				
2	**Background**		Explain the scientific background and rationale for the reported study. What is the exposure? Is a potential causal relationship between exposure and outcome plausible? Justify why MR is a helpful method to address the study question	✓ pp. 4, 5	
3	**Objectives**		State specific objectives clearly, including pre-specified causal hypotheses (if any). State that MR is a method that, under specific assumptions, intends to estimate causal effects	✓ pp. 4, 5	
	**METHODS**				
4	**Study design and data sources**		Present key elements of the study design early in the article. Consider including a table listing sources of data for all phases of the study. For each data source contributing to the analysis, describe the following:	✓ For a)-e) STables 2-4 and references provided	
		a)	Setting: Describe the study design and the underlying population, if possible. Describe the setting, locations, and relevant dates, including periods of recruitment, exposure, follow-up, and data collection, when available.		
		b)	Participants: Give the eligibility criteria, and the sources and methods of selection of participants. Report the sample size, and whether any power or sample size calculations were carried out prior to the main analysis	Power calculations were not carried out	
		c)	Describe measurement, quality control and selection of genetic variants		
		d)	For each exposure, outcome, and other relevant variables, describe methods of assessment and diagnostic criteria for diseases		
		e)	Provide details of ethics committee approval and participant informed consent, if relevant		
5	**Assumptions**		Explicitly state the three core IV assumptions for the main analysis (relevance, independence and exclusion restriction) as well assumptions for any additional or sensitivity analysis	✓ pp. 4, 5	

6	**Statistical methods: main analysis**		Describe statistical methods and statistics used	
		a)	Describe how quantitative variables were handled in the analyses (i.e., scale, units, model)	✓ p. 6, 11
		b)	Describe how genetic variants were handled in the analyses and, if applicable, how their weights were selected	Referenced
		c)	Describe the MR estimator (e.g. two-stage least squares, Wald ratio) and related statistics. Detail the included covariates and, in case of two-sample MR, whether the same covariate set was used for adjustment in the two samples	✓ pp. 9-10
		d)	Explain how missing data were addressed	Missing SNPs are detailed and surrogates described
		e)	If applicable, indicate how multiple testing was addressed	N/A
7	**Assessment of assumptions**		Describe any methods or prior knowledge used to assess the assumptions or justify their validity	Sensitivity analyses are shown in Tables 1 and 2. No prior knowledge used
8	**Sensitivity analyses and additional analyses**		Describe any sensitivity analyses or additional analyses performed (e.g. comparison of effect estimates from different approaches, independent replication, bias analytic techniques, validation of instruments, simulations)	✓ p. 12
9	**Software and pre-registration**			
		a)	Name statistical software and package(s), including version and settings used	✓ p.10
		b)	State whether the study protocol and details were pre-registered (as well as when and where)	N/A
	**RESULTS**			
10	**Descriptive data**			
		a)	Report the numbers of individuals at each stage of included studies and reasons for exclusion. Consider use of a flow diagram	references provided
		b)	Report summary statistics for phenotypic exposure(s), outcome(s), and other relevant variables (e.g. means, SDs, proportions)	references provided

		c)	If the data sources include meta-analyses of previous studies, provide the assessments of heterogeneity across these studies	references provided
		d)	For two-sample MR:<br>i. Provide justification of the similarity of the genetic variant-exposure associations between the exposure and outcome samples<br>ii. Provide information on the number of individuals who overlap between the exposure and outcome studies	
11	**Main results**			
		a)	Report the associations between genetic variant and exposure, and between genetic variant and outcome, preferably on an interpretable scale	✓ STables allANMinput_270225.xlsx<br>allAAMinput_270225.xlsx
		b)	Report MR estimates of the relationship between exposure and outcome, and the measures of uncertainty from the MR analysis, on an interpretable scale, such as odds ratio or relative risk per SD difference	✓ Tables 1 and 2
		c)	If relevant, consider translating estimates of relative risk into absolute risk for a meaningful time period	N/A
		d)	Consider plots to visualize results (e.g. forest plot, scatterplot of associations between genetic variants and outcome versus between genetic variants and exposure)	✓ Figures 2-4
12	**Assessment of assumptions**			
		a)	Report the assessment of the validity of the assumptions	✓ p.12-17
		b)	Report any additional statistics (e.g., assessments of heterogeneity across genetic variants, such as I^2, Q statistic or E-value)	✓ p.12
13	**Sensitivity analyses and additional analyses**			
		a)	Report any sensitivity analyses to assess the robustness of the main results to violations of the assumptions	As above
		b)	Report results from other sensitivity analyses or additional analyses	Radial MR
		c)	Report any assessment of direction of causal relationship (e.g., bidirectional MR)	N/A

		d)	When relevant, report and compare with estimates from non-MR analyses	Data not available
		e)	Consider additional plots to visualize results (e.g., leave-one-out analyses)	N/A
	**DISCUSSION**			
14	**Key results**		Summarize key results with reference to study objectives	✓ p.13-17
15	**Limitations**		Discuss limitations of the study, taking into account the validity of the IV assumptions, other sources of potential bias, and imprecision. Discuss both direction and magnitude of any potential bias and any efforts to address them	✓ p.17
16	**Interpretation**			
		a)	Meaning: Give a cautious overall interpretation of results in the context of their limitations and in comparison with other studies	✓ p. 13-17
		b)	Mechanism: Discuss underlying biological mechanisms that could drive a potential causal relationship between the investigated exposure and the outcome, and whether the gene-environment equivalence assumption is reasonable. Use causal language carefully, clarifying that IV estimates may provide causal effects only under certain assumptions	✓ p. 13-17
		c)	Clinical relevance: Discuss whether the results have clinical or public policy relevance, and to what extent they inform effect sizes of possible interventions	✓ p. 17
17	**Generalizability**		Discuss the generalizability of the study results (a) to other populations, (b) across other exposure periods/timings, and (c) across other levels of exposure	✓ p. 17
	**OTHER INFORMATION**			
18	**Funding**		Describe sources of funding and the role of funders in the present study and, if applicable, sources of funding for the databases and original study or studies on which the present study is based	✓ p.20, 21
19	**Data and data sharing**		Provide the data used to perform all analyses or report where and how the data can be accessed, and reference these sources in the article. Provide the statistical code needed to reproduce the results in the article, or report whether the code is publicly accessible and if so, where	Referenced<br>Code can be provided on request
20	**Conflicts of Interest**		All authors should declare all potential conflicts of interest	✓ p.20

This checklist is copyrighted by the Equator Network under the Creative Commons Attribution 3.0 Unported (CC BY 3.0) license.

1. Skrivankova VW, Richmond RC, Woolf BAR, Yarmolinsky J, Davies NM, Swanson SA, et al. Strengthening the Reporting of Observational Studies in Epidemiology using Mendelian Randomization (STROBE-MR) Statement. JAMA. 2021;under review.

2. Skrivankova VW, Richmond RC, Woolf BAR, Davies NM, Swanson SA, VanderWeele TJ, et al. Strengthening the Reporting of Observational Studies in Epidemiology using Mendelian Randomisation (STROBE-MR): Explanation and Elaboration. BMJ. 2021;375:n2233.
